# Preferences of health professions learners: a scoping review of the use and role of DCEs in health professions education

**DOI:** 10.1080/10872981.2026.2614235

**Published:** 2026-01-13

**Authors:** Sarah O'Neal, Natalie Smith, Jan Ostermann, Binbin Zheng, Laiton Steele, Chris Gillette

**Affiliations:** a Department of PA Studies, Wake Forest University School of Medicine, Winston-Salem, NC, USA; b Arnold School of Public Health, University of South Carolina, Columbia, SC, USA; c Department of Health Professions Education, Uniformed Services University of the Health Sciences, Bethesda, MD, USA; d Department of Epidemiology and Prevention, Wake Forest University School of Medicine, Winston-Salem, NC, USA

**Keywords:** Economics, medical, nursing education research, education, professional, education, pharmacy, education, medical, stated preferences

## Abstract

While discrete choice experiments (DCE), have increasingly been used in the medical literature, little is known about the use of these methods for eliciting preferences from and about students and trainees in health professions. The objectives of this scoping review are to (1) describe the extent to which DCEs have been used in health professions’ education, (2) identify which health professionals have been studied, and (3) identify thematic areas of research in which these methods have been used thematic areas. Between June and September 2024, we conducted a scoping review of the PubMed/Medline, EconLit, Web of Science, and Global Index Medicus databases to identify articles. Studies were eligible for inclusion in this review if they included a health profession training population and if conjoint analysis, DCE, or best-worst scaling studies were used. Forty-nine articles, comprising 60 studies and 21,731 health profession trainees, were included in this review. Medical and nursing students constitute the majority of the population studied. The greatest number of studies have been conducted in China (*n* = 11) and the United States (*n* = 8). The two most popular thematic areas in which these studies have been used are to identify preferences for policies and incentives to take a job in a rural area and residency training preferences for medical students. There has been a gradual increase in the use of these methods in the health profession education literature. The extent to which findings have been used for curriculum or policy design is not clear. DCEs are increasingly used to study health profession students and other trainees. More research is needed to explore the validity of preferences and whether preferences correlate with student outcomes or observed behavior.

## Introduction

Discrete choice experiments (DCE) are increasingly used in health services research as a means to obtain preferences for health services or therapeutics [1–3]. Econometric approaches such as conjoint analysis, DCEs, and best-worst scaling experiments are powerful methods for identifying preferences for a service or product and for characterizing value and trade-offs within a given population [2]. These methods, created in the field of marketing, can be easily adapted for use in a variety of research questions in which preferences drive decision-making [4]. Examples include pharmaceutical research to identify patient preferences for certain pharmacological characteristics (e.g., tolerability, side effects) to facilitate shared decision-making [1]. Another example is the use of methodology to identify preferences for policies and incentives to increase physician recruitment and retention in rural areas or for how to discuss deprescribing with older patients [5,6]. All of these methods have an underlying central premise that consumer preferences drive real-world choices.

Conjoint analysis, DCE, and best-worst scaling experiments all share the goal of eliciting preferences. Conjoint analyzes are used to identify product or service characteristics that individuals value. DCEs are used to identify and predict behavioral choices among two or three competing alternatives [4]. Best-worst scaling experiments are a type of DCE where participants are asked to select the best and worst attribute levels to identify the most important attribute [7]. DCE methods are based on two interconnected theoretical underpinnings: Lancaster’s consumer theory (LCT) and random utility theory (RUT) [4,8]. LCT states that the combination of good or service characteristics (attributes) gives rise to value, or utility. The RUT states that utility is a latent variable and that when an individual chooses a good or service, its utility at least matches or exceeds the utility of the other alternatives. According to the RUT, consumer behavior has a systematic and explainable component along with a random (error) component. These methods use surveys and ask respondents to choose between competing alternatives that differ from each other based on their attributes [5]. Choices are analyzed to identify the relative value of each attribute and which trade-offs individuals are willing to make between different attributes [8]. In addition to identifying preferences, the DCE methodology allows for the evaluation of hypothetical policy options and the prospective design of targeted, preference-concordant policies without the cost of iterative implementations and adaptations and lag times needed to observe the impact (on choices) resulting from policy change [9]. Under the flexible RUT paradigm, DCEs allow researchers to target specific policies to specific groups of people based on latent class analysis based on stated preferences.

To our knowledge, few studies have focused on DCEs in health professions education (HPE) journals, and few educators are aware of this methodology [10]. Identifying learners’ preferences can help educators and administrators target specific curricular areas for improvement to increase student satisfaction. Studying learners' preferences also has the potential to improve future aspects of access to and quality of care. We sought to conduct a scoping review of the health professions education literature to better understand how these methods have been used, in what student/trainee populations they have been used, and in what contexts these methods have been used. We also sought to identify knowledge and gaps in the literature regarding the use of these methods in health professions. The purpose of this scoping review, therefore, was to (1) describe the extent to which discrete choice experiments have been used in health professions’ education, (2) identify which health professionals have been studied, and (3) identify thematic areas of research in which these methods have been used. Accomplishing these aims allows for a more comprehensive mapping of a research agenda for DCEs in health professional training contexts.

## Methods

### Protocol development and search strategy

This scoping review was developed using the Preferred Reporting Items for Scoping reviews-Scoping Review (PRISMA-ScR) checklist (Appendix A) [11]. This scoping review was registered on the Open Science Framework review registry (D6B9G) [12]. We searched the PubMed/MEDLINE, EconLit, Web of Science, and Global Index Medicus (GIM) databases. The GIM database covers literature from the western Pacific (WPRIM), Latin America and the Caribbean (LILACS), the eastern Mediterranean (IMEMR), Southeast Asia (IMSEAR), and Africa (AIM), which are all traditionally underrepresented in ‘global north’ journals [13]. We did not include Embase or Scopus in the searched databases because of substantial overlap with PubMed/Medline and Web of Science, per our experience and other reviews [1]. Because we did not set out to complete a systematic review, it was not our intention to find all DCEs but to identify the scope of their use in HPE. The preliminary searches, piloting, and full searches occurred between June 2024 and October 2024. The search strategies for all the databases are presented in Appendix B. There were no date or language restrictions. The last search date was April 23, 2025.

### Inclusion criteria and article selection

The research questions this scoping review addressed were as follows: (1) To what extent have health profession educators utilized DCEs in health profession education research?; (2) In what health profession trainee populations have these methods been used?; and (3) in what thematic areas have these methods been used? To operationalize the questions into the review objectives, we included articles that had (1) studied medical, pharmacy, nursing, physician assistant (PA)/clinical officers (CO), nurse practitioners (NP), occupational therapy (OT), and physical therapy (PT) students as well as postgraduate medical trainees (e.g., junior doctors and medical residents) and (2) used a quantitative, stated preferences design including (a) conjoint analysis, (b) DCE, or (c) best-worst scaling experiment. We excluded other quantitatively stated preference methods (e.g., rating and ranking) to ensure a focus on conjoint analysis, DCEs, and best-worst scaling experiments. We also excluded study protocols and systematic/scoping/narrative reviews on the methodologies used to accomplish the study’s objectives. We excluded conference proceedings and abstracts to ensure that we could answer the research questions. To maintain our focus on clinical health profession learners, we excluded articles that included only public health professionals, research doctorate (i.e., PhD) students, or any nonpatient-facing health professional students. Two authors (CG, SG) independently screened the articles’ titles and abstracts for inclusion. If there was disagreement on whether the article should be included, both authors met and resolved the disagreement by consensus. A third author (NS) was used to break the tie in case of failure to reach agreement. We utilized Covidence (Melbourne, Australia) for title/abstract screening and full-text inclusion.

### Data extraction and study quality

Two authors extracted information from the included articles (CG and BZ). We extracted the following information: (1) article title, (2) lead author contact details, (3) year published, (4) country in which the study was conducted, (5) the type of health professional studied, (6) the methods used, (7) the research question and choice context, (8) total number of participants, (9) the studied attributes and attribute levels, (10) the number of choice sets, (11) the analytical method, and (12) findings from the primary and secondary outcomes, if applicable.

Two authors (CG and LS) used an inductive approach to identify the thematic areas in which the studies were conducted. First, we examined the health professional population(s) that were being studied. Second, we identified the outcome in which the study was being used to provide evidence. This involved identifying the choice context, if provided, and using the primary objectives and outcomes to identify the thematic area. Each author independently conducted thematic analysis. We resolved discrepancies through discussion and consensus.

### Data synthesis

The principal goals of this review were to identify in what situations DCE and DCE-like study designs have been used in health professional education and to identify what outcomes were studied when DCE strategies were used. Two authors (CG and LS) examined each study’s theme based on the research question and choice context. The authors independently coded each study’s theme and then compared the findings. Discrepancies were resolved via consensus. We qualitatively synthesized the situations in which DCEs were used as well as the outcomes studied.

## Results

**Figure 1.**
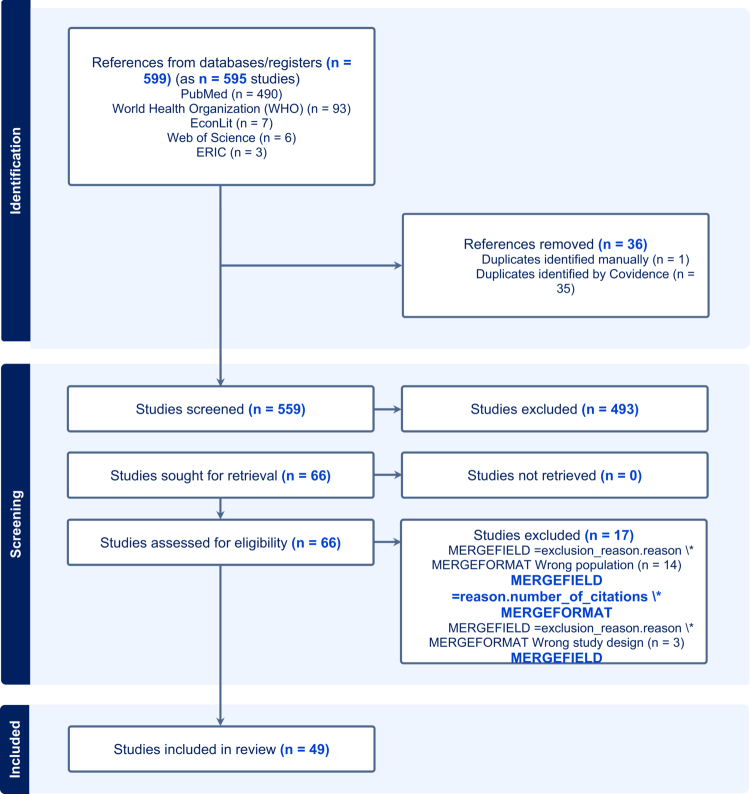
PRISMA-ScR diagram.

Figure 1 presents the PRISMA diagram showing the flow chart. Our search strategy retrieved 599 references, and we screened 559 articles. Next, we assessed 66 full-text articles and ultimately included 49 articles (*n* = 60 individual studies) of 21,731 students in this review.

**Figure 2.**
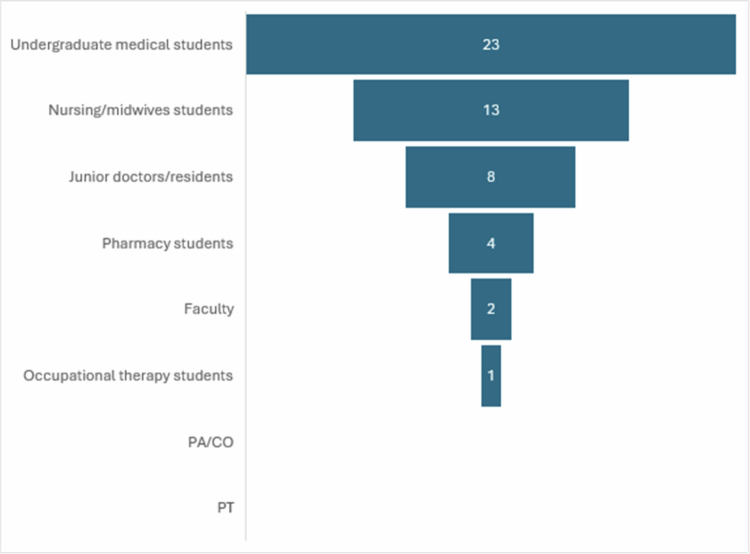
Health professional trainees studied to date.

**Figure 3.**
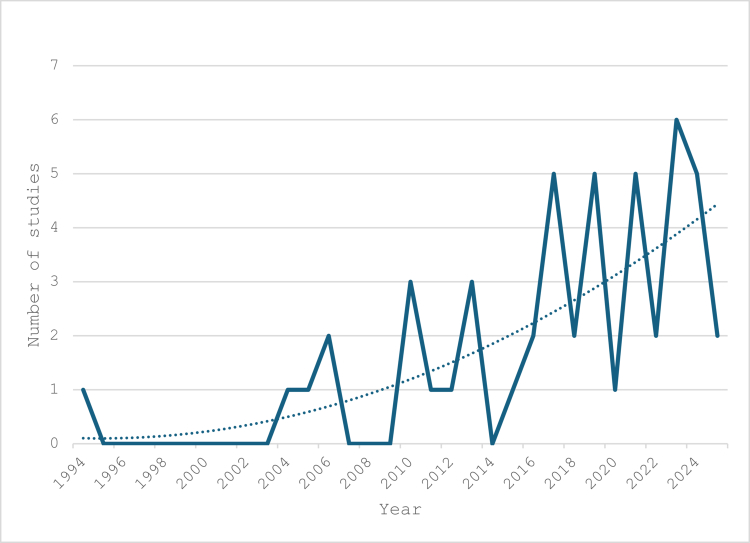
Use of DCEs in health professions education over time.

**Figure 4.**
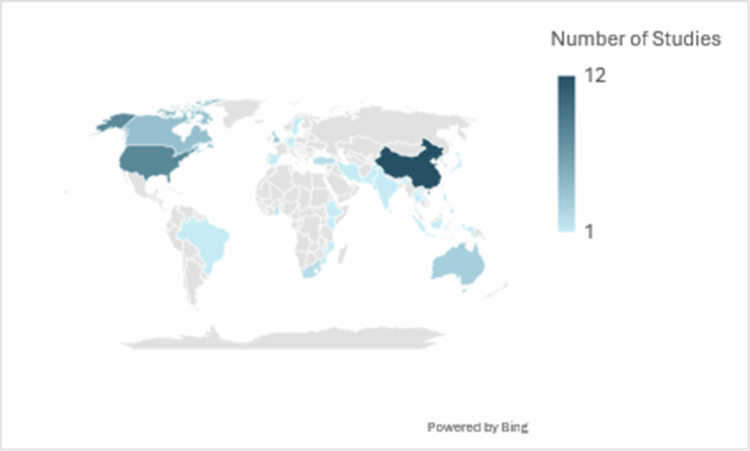
Use of DCEs globally.

Table 1 presents the characteristics of the included studies. The greatest number of articles studied undergraduate medical students (*n* = 22) and nursing students (*n* = 12) (Figure 2). The year with the highest number of publications occurred in 2022 (*n* = 6). There is a general trend of increasing use of these methods in the HPE literature (Figure 3). The country with the greatest number of publications to date is China (*n* = 11), and the country with the next greatest number of publications is the United States (*n* = 8) (Figure 4). The journal with the greatest number of publications was *BMC Medical Education* with 9 articles.

**Table 1.** Characteristics of included articles.

Study ID	Country in which the study conducted	Aim of study	Study Design	Population description	Total number of participants	Number of studies	Number of attributes
Diamond 1994 [14]	United States	Identify preferences for medical specialties	CA	Final year medical students	104	1	6
Tsang 2004 [15]	China	Identify factors that influence OT students' attitudes toward persons with disabilities	CA	Occupational therapy student	108	1	5
Thompson 2005 [16]	UK	Identify changes in information after a lecture	CA	Student nurses taking a degree level educational module in critical care	23	1	6
Cunningham 2006 [17]	Canada	Identify students' preferences for a redesigned small group, problem-based undergraduate medical education program	DCE	Undergraduate medical students in years 1–4	254	1	14
Izumi 2006 [18]	Japan	Identify preferences for clinical training hospitals by medical interns	CA	Physicians	46	1	8
Grindrod 2010 [19]	Canada	Preferences for providing patient-centered services	DCE	Pharmacy	59	1	6
Kruk 2010 [20]	Ghana	Identify specific job attributes that influenced medical students' stated preference for hypothetical rural job postings	DCE	Medical students	302	1	7
Blaauw 2010 [21]	Kenya, South Africa, Thailand	Recruitment of nurses to rural areas	DCE	Final year nursing students	1064	3	7
Wang 2011 [22]	Canada	Identify preferences for ranking residency programs	BWS	Medical students	339	1	13
Sivey 2012 [23]	Australia	Identify preferences for specialty choice among junior doctors	DCE	Junior doctors	536	1	7
Ageyi-Baffour 2013 [24]	Ghana	Recruit midwifery students to rural and underserved areas	DCE	Final year midwifery students	238	1	7
Rao 2013 [25]	India	Identify preferences for policies to increase recruitment to rural areas	DCE	Final year medical and nursing students	293	2	8
Honda 2015 [26]	Mozambique	Identify preferences for policies and incentives to recruit non-physician health professionals to rural areas	DCE	Mid-level specialists (nurses, midwives, etc.)	123	1	8
Liang 2016 [27]	China	Preferences for PCP-based specialty choices	BWS	Medical students	190	1	11
Efendi 2016 [28]	Indonesia	Analyze job preferences to identify effective policies to improve recruitment and retention of health students in remote areas	DCE	Final year medical students, nursing students, and midwifery students	400	3	6
Girardi 2017 [29]	Brazil	Identify job preferences to recruit to medically underserved areas	DCE	Final year medical students	277	1	6
Keuffell 2017 [30]	Lao PDR	Identify most cost-effective incentive packages to recruit medical students to rural areas	DCE	Fifth-year, sixth-year, and post-graduate medical students in family medicine	329	1	6
Factor 2017 [31]	Philippines	Preferences of student nurses' preferences of clinical instructors	CA	Junior and senior nursing students at one college of nursing	227	1	4
Ramos 2017 [32]	Portugal	Identify determinants of medical specialty and residency placement	DCE	Junior doctors in Portugal	520	1	8
Cleland 2017 [33]	UK	Examine career preferences and relative strength of those preferences in final-year medical students	DCE	Final year medical students	761	1	6
Liu 2018 [34]	China	Stated preferences for jobs to address maldistribution of physicians in China	DCE	Medical students	519	1	6
Scanlan 2018 [35]	UK	Identify characteristics of training posts for residency	DCE	FP2 doctors in Scotland	677	1	6
Liu 2019 [36]	China	Explore job preferences of undergraduate nursing students	DCE	Nursing students	507	1	6
Macindo 2019 [37]	Philippines	To elucidate acute & critical care experiential learning preferences of student nurses	DCE	Undergraduate student nurses	213	1	5
Rankin 2019 [38]	Sweden, Australia	Preferences for a chronic pain condition	BWS	Medical students in Sweden and Australia	110	2	11
Ulrich 2019 [13]	United States, Canada	Identify preferences for how much additional salary would be necessary for students to choose a position in different practice and geographic settings in the central US and Canada	DCE	Pharmacy students	283	2	5
Sawarynski 2019 [39]	United States	Evaluate Embark program at Oakland University medical school	CA	Undergraduate medical students	367	1	3
Scanlan 2020 [40]	UK	What is the difference between male and female doctors when entering residency/specialty training in the United Kingdom	DCE	Junior doctors	5005	1	6
Yoo 2020 [41]	Australia	Comparing results of a multi-profile BWS and a profile case BWS	BWS	Bachelor degree nursing students and new graduates	526	2	12
Bao 2021 [42]	China	Identify specific incentives to recruit physicians and nurses to rural areas	DCE	Medical and nursing students	787	1	8
Liu 2021 [43]	China	Investigate relative importance of attributes influencing job preferences of undergraduate pharmacy students in mainland China.	DCE	Pharmacy students	581	1	6
Meng 2021 [44]	China	Identify preferences for working in geriatrics	DCE	Final year master's degree nursing students	267	1	6
Noben 2021 [45]	Netherlands	Ascertain what attributes are important when teaching value-based healthcare	DCE	Medical residents	197	1	6
Maxfield 2021 [46]	United States	Determine changes in relative importance of resident application attributes when numerical Step 1 results are replaced.	DCE	Faculty in radiology departments	202	1	9
Kiyak 2022 [47]	Turkiye, Spain, and Pakistan	What are differences between preferences for specialty training for medical students in Turkiye, Spain, and Pakistan?	DCE	Medical students	491	3	7
Wu 2022 [48]	United States	Explore work-stressor rankings and preferences for wellness interventions	BWS	Medical residents	267	1	20
Tian 2023 [49]	China	Job preferences of preventive medicine students in China during COVID19 pandemic	DCE	Preventive medicine students	307	1	6
Yao 2023 [50]	China	Identify preferences for case-based learning in undergraduate nursing students	DCE	Undergraduate nursing students	227	1	6
Zhang 2023 [51]	China	Identify how altruism impacts heterogeneity of job preferences	DCE	Medical students	741	1	6
Engidaw 2023 [52]	Ethiopia	Identify preferences for rural practice features and predict rural job uptake	DCE	Medical students	352	1	6
Jose 2023 [53]	South Africa	Describe relative valuation of rural hospital characteristics among final-year medical students	DCE	Medical students	193	1	7
Perez 2023 [54]	United States	What attributes of equitable assessment in clinical training are most valued by medical trainees	DCE	Medical students	306	2	6
Xin-Yan 2024 [55]	China	To investigate the work location choices of medical students from different demographics, i.e., birthplaces	DCE	Medical students	741	1	6
Vomhof 2024 [56]	Germany	Explore information preferences for digital mental health interventions amongst medical students.	DCE	Medical students	231	1	5
Tozduman 2024 [57]	Turkiye	Identify preferences for jobs in rural area	DCE	Medical students	102	1	6
Carr 2024 [58]	United States	Understand reasons for decisions about work-hour restrictions among medical residents.	DCE	Medical residents	113	1	6
Maxfield 2024 [59]	United States	Examining the influence of extracurricular activities on radiology resident selection	DCE	Faculty in radiology departments	244	1	9
Guo 2025 [60]	China	Investigate Chinese undergraduate pharmacy students' preferences for case-based learning	DCE	Undergraduate pharmacy students	482	1	6
Moradi 2025 [61]	Iran	Investigate hospital selection preferences of nursing students.	DCE	Undergraduate nursing students at Kermanshah University of Medical Sciences, western Iran	500	1	6

Abbreviations: DCE-discrete choice experiment, BWS-best-worst scaling experiment, CA-conjoint analysis.

### Thematic areas in which HPE DCE methods used

**Figure 5.**
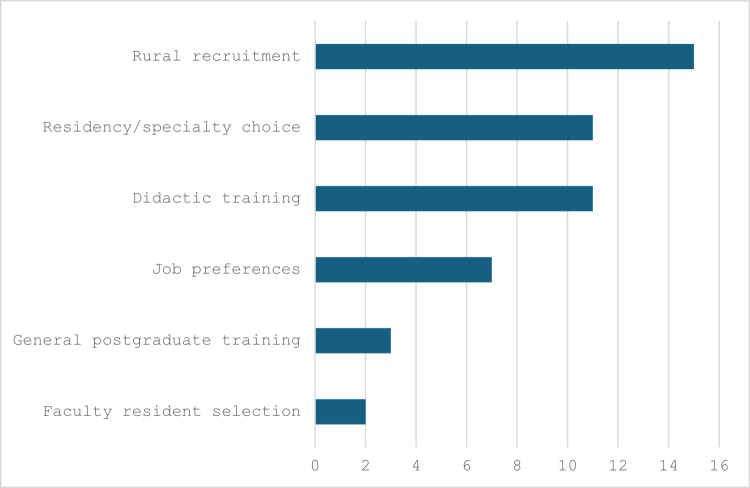
Thematic areas in Which DCEs have been used in health professions training.

The greatest number of articles in which DCE methods have been used to date in HPE have focused on job preferences with a focus on recruiting health professionals to rural areas (*n* = 16). Preferences in didactic training were the second most commonly studied context (*n* = 9). Figure 5 presents the distinct thematic areas in which DCEs have been used in HPE and the frequencies for each thematic area.

### Context 1-Job preferences: recruiting health professionals to rural areas

Fifteen (*n* = 15) articles investigated health profession learners’ preferences for taking a job in rural areas [20,21,24–26,28–30,34,36,42,43,49,52,56]. Fourteen of the 15 studies found that there was an aversion to working in a rural area in pooled analyzes. However, some respondents preferred rural jobs. South African nurses had a greater preference for rural clinics, whereas Kenyan and Thai nurses preferred more urban areas [28]. Those who were born in rural areas had less aversion to working in rural areas. Blaauw et al. found that the effect of being born in a rural area among South African, Kenyan, and Thai nursing students was similar to a 10% salary increase for taking a job in a rural area [28].

Two (*n* = 2) studies examined preference heterogeneity in job choices for rural areas. Both studies revealed substantial heterogeneity among the subgroups. For example, nursing students in Kenya and South Africa preferred higher salaries, whereas Thai nursing students preferred additional benefits [28]. In one study in Indonesia that examined medical students, nursing students, and midwifery students, medical students most preferred full tuition for future study, nurses preferred higher salaries, and midwifery students most preferred having an advanced facility [25].

In all studies conducted in China (*n* = 5), medical students most preferred higher salaries for jobs in rural areas [29,34,42,43,49]. Examining the regression coefficients from each study provides important nuance that is important to mention: because salaries are measured as a continuous variable, the regression coefficients are quite small (e.g., 0.0003); however, when scaled to a policy-level impact (e.g., thousands of CNY), the salary coefficients in these studies match or exceed preferences for nonmonetary attributes. For example, in Bao and colleagues’ study of medical and nursing students’ preferences for employment in rural China, increasing salary by 1,443 CNY would exceed the preference for having a permanent contract [49]. One study in Brazil also found that final-year medical students preferred higher salaries to work in rural areas [56].

Among the nonmonetary attributes, five studies found that health profession trainees preferred additional training and specialization opportunities [20,21,24,28,52]. Other nonmonetary attributes that were found to increase acceptance of a job in a rural area include providing housing and transportation and patient care opportunities [26].

### Context 2-Didactic training

Eleven studies investigated health profession students’ preferences for didactic training [15–17,23,31,37–39,50,54,60]. Three studies examined problem-based and case-based learning preferences, one in Canada and two in China [15,17,39]. All of these studies examined student group size and found conflicting results. In the study by Cunningham, the analysis revealed two latent classes of Canadian medical students: one group (*n* = 218) preferred the smallest group size (5 per group), whereas the other latent class (*n* = 36) preferred larger groups of 8 students per group [15]. Yao et al. found that nursing students preferred a group size of 9–11 students per group over alternate group options of 4–6 students and 14–16 students [17]. Guo et al. found that undergraduate pharmacy students in China preferred the smallest group size [39]. While the nursing students in Yao’s study had the greatest preference for group size, they also preferred the case to be presented by video, but the pharmacy students in Guo et al. preferred the role play format the most [17,39]. The Canadian study revealed that the modality was of lower importance than the tutorial group size in both latent classes; however, both groups preferred in-person simulated patients and computerized simulations [15].

Two studies examined nurses’ preferences in critical care training experiences [31,38]. Thompson et al. used the CA approach to examine nursing students’ preferences for clinical information in the United Kingdom [38]. The findings revealed that clinical training impacted how nursing students valued certain types of clinical information, increasing value for some attributes (e.g., respiratory rate) and decreasing value for others in hypovolemic shock cases (e.g., pulse). Macindo and colleagues used the DCE approach to study undergraduate nursing students’ preferences for experiential training in acute and clinical settings [31]. The findings indicated that nursing students most preferred to spend at least one week (21 hours) per unit rather than 7 hours per unit, differing significantly by nursing student gender.

The other studies’ didactic choice contexts differed substantially, preventing any meaningful synthesis. Tsang, Chan, and Chan examined occupational therapy students’ attitudes toward people who have disabilities in Hong Kong [23]. Factor and B. de Guzman studied nursing students’ preferences for their clinical instructors [50]. Sawarynski and Baxa studied medical students’ evaluations of research curriculum modules using conjoint analysis [54]. Rankin and colleagues examined Swedish and Australian medical students’ preferences for chronic pain management and identified the importance of factors that influence chronic pain management [37]. Perez et al. studied preferences to ensure equitable assessment during clerkships [16], and Vomhof et al. investigated medical students’ preferences for digital mental health interventions [60].

### Context 3-Postgraduate training: specialty selection

Eleven studies examined specialty selection or specialty location selection in medical students or residents [14,18,22,27,32,33,35,40,47,53,57]. There were 3 identified subthemes in this context plus one study that did not fit into one of the 3 themes. The first subtheme evaluated choosing between primary care specialties and surgical or subspecialty residencies (*n* = 3) [22,47,53]. Ramos and Sivey both studied junior doctors’ preferences for specialties while Liang studied medical students. Both Ramos and Sivey investigated salary as one option to increase the number of trainees to choose primary care specialties, and in both studies, higher salaries were preferred over lower salaries [22,53]. Liang conducted a best-worst scaling study that did not include salary; medical students in this study in China had the greatest preference for general surgery [47]. However, primary care internal medicine was chosen as the second most preferred specialty over OB/GYN, pediatrics, and other specialties. Ramos and Sivey also found other attributes that might also improve trainees’ ability to choose general practices [22,53]. Ramos found that providing opportunities for Portuguese junior doctors to work in the private sector increased the probability of choosing a specific job [53]. Sivey et al. found that increasing opportunities for medical procedures and research might increase the number of trainees choosing to work in general practice [22].

Three studies examined preferences for specialty training. Kiyak and Diamond both found that the personal perspectives of specialties impact the willingness to choose a specialty [18,40]. Kiyak found this finding for medical students in both Turkey and Spain, whereas it was not important in Pakistan [40]. In a study of medical students in the United States, Diamond found that personal perceptions of a specialty were chosen most often in a conjoint analysis study [18].

Four studies examined preferences for choosing postgraduate training programs [14,27,32,33]. Cleland and Wang both found that resident morale and having a supportive working culture were the most preferred attributes in choosing a postgraduate training program [27,32]. Wang and colleagues found that having a variety of clinical experiences was the most preferred attribute for Canadian medical students in choosing a residency program [27]. Scanlan compared gender differences in preferences for specialty training posts of Foundation Program Year 2 (F2) doctors [14]. This study revealed that, similar to studies on general job preferences, females preferred a supportive culture more than males did. Another study by Maeta and Minowa reported that learners in Japan valued having a famous teaching doctor as the most preferred attribute [33].

Finally, one study investigated preferences for a rural internship in South Africa [57]. Jose and colleagues found that medical students had the greatest average preference for performing more procedural work compared to completing forms did.

### Context 4-General job preferences (without rural preferences)

Seven articles evaluated the general job preferences of health profession learners [19,41,44,51,55,58,61]. Three articles studied nursing students [51,55,58], 3 studied medical students [19,44,61], and 1 studied pharmacy students [41]. In almost every case, salary was viewed as most important for their job. While salary was viewed as most important for job selection, other factors also significantly impacted job preferences. In Grindrod et al.’s study of Canadian pharmacy students, latent class analysis revealed that one profile (50% of the sample) valued service type (e.g., medication therapy management versus typical pharmacy services) more than any other attribute [41]. The other profile (50% of the sample) valued salary the most. Zhang and colleagues found that a higher salary was most valued among Chinese medical students; however, further analysis revealed that those with higher intrinsic altruism valued their salary less than those with less altruism did, as evidenced by the negative *β* coefficient in the mixed logit model [61]. In two of three studies of nursing students, nursing students valued working conditions and supportive management the most in China [51] and Australia [58], whereas those from Iran most valued salary [55]. Crucially, none of these studies evaluated whether there were any gender differences in altruism, salary preferences or preferences for nonfinancial attributes (e.g., workload). Xin-Yan et al. found that those who were born or raised in rural areas may have different job preferences than those who were from urban areas (e.g., career development opportunities) [19].

### Context 4-Postgraduate training: general

Three articles investigated general postgraduate training preferences among medical residents [45,46,48]. Two of the studies were conducted in the US [45,48], while one was conducted in the Netherlands [46]. Wu and colleagues investigated the well-being and stress levels of medical residents from one institution in the northeast United States [45]. Residents reported that difficulty balancing work and life demands was the greatest stressor they encountered. The study also investigated preferences for wellness interventions and revealed that residents preferred therapy the most, followed by coaching, which was the second-most preferred workplace wellness intervention. Carr and colleagues investigated why residents decide to work during their work-hour restriction time, which is mandated by the Accreditation Council for Graduate Medical Education (ACGME) [48]. Residents from one institution in the South Atlantic region of the US completed an online DCE. This study revealed that positive attending physician feedback had the greatest influence on choosing to take a postcall day off, whereas when an attending physician did not give positive feedback or spoke negatively about residents who took a postcall day off, it had the greatest influence on choosing to work during work-hour restrictions.^58^Noben and colleagues investigated residents’ preferences regarding how to be educated about value-based medical care in the southeastern Netherlands [46]. The study revealed that the attribute that residents placed the greatest preference for was knowledge combining medical practice and process of care and had the greatest aversion to learning about value-based care outside of work (e.g., during leisure time) [46].

### Context 5-Faculty resident selection

Two articles investigated faculty preferences for selecting postgraduate residents from 14 radiology postgraduate programs [59,62]. These were also the only two studies that investigated medical school faculty preferences. In study 1, faculty who selected medical residents at 14 residency programs located throughout the United States completed an online DCE to examine whether preferences in selecting medical residents changed as a result of the Step 1 exam changing from a numerical score to a Pass/Fail system [59]. When the Step 1 score was presented numerically, the participants placed the greatest preference on medical school attendance, race/ethnicity, and Step 1 score. When Step 1 was presented as pass, the results were the same, except that the Step 2 score replaced the Step 1 score as the third greatest preference. The participants placed even more emphasis on which medical school the resident attended when the Step 1 score was presented as pass, whereas the preference for race/ethnicity decreased in importance. In Study 2, faculty from 30 postgraduate radiology programs evaluated hypothetical residents using similar attributes and levels similar to those used in Study 1 but included several extracurricular activities [62]. The study revealed that participating in more extracurricular activities was preferable to participating in fewer extracurricular activities. However, because the attributes that participants presented varied owing to personal preferences, it was impossible to identify any specific extracurricular activities that most influenced resident selection.

## Discussion

This is the first scoping review of DCE studies in the HPE literature. A recent review noted significant growth in health-related DCEs since the early 2000s. Our study revealed a similar trend in HPE, particularly in examining medical and nursing trainees’ preferences for rural practice. The wide variety of thematic areas shows the method's flexibility and potential for future HPE studies in which there are preference-sensitive decisions. We found that the majority of DCE research in health professions education has used the DCE method, with limited examples of conjoint analysis or best-worst scaling experiments.

Our findings yield several significant implications for researchers in HPE. First, the literature on DCEs in HPE is predominantly focused on medical students and nurses, with limited representation of other health profession trainees, such as those in pharmacy, PA/CO, and PT programs. This indicates a gap in the literature regarding the educational preferences of these groups of trainees. This is an important gap in the literature because these professions are experiencing similar issues as medicine and nursing (e.g., declining numbers of clinicians in rural areas and postgraduate residencies). Aligning policies and incentives to increase access to these professionals for patients is needed, which can generally be better understood only by obtaining these clinicians’ preferences. Professions in which there are no or limited DCEs present an exciting challenge and could uncover hidden preferences and help set new standards for their respective professions [63–65]. Second, the existing HPE DCE literature is geographically narrow, with only a few countries being represented, highlighting the need for a broader international perspective in future research.

DCEs are a powerful tool to elicit preferences and trade-offs among health profession trainees and faculty. DCEs can identify teaching methods that students value most; for example, if students trade off lecture time for active learning exercises or simulation, curriculum designers can adjust the classroom and activity time to ensure that simulation occurs more frequently to facilitate student learning. If students prefer more time in active learning, then administrators can provide faculty development and guidance on how to effectively design classroom time to devote to active learning instead of lectures. DCEs can also uncover students’ preferences for assessment and feedback, allowing educators to design effective assessment strategies. However, since educational institutions are required to align with accreditation requirements and community priorities, DCEs offer only one form of input regarding education and assessment.

Conjoint analysis, DCE, and best-worst scaling experiments offer distinct methodological advances over other types of stated preference methods, such as rating and ranking or Likert-type answer scales. With rating and ranking type items, participants can and often do rank product or service characteristics as having equal importance. With Likert-type answer scales, participants can give different characteristics the same ‘score’. Conjoint analysis, DCEs, and best-worst scaling experiments force participants to identify their preferences and differentiate the relative importance of attributes, whereas other stated preference designs cannot [66].

### Future research agenda

A critical takeaway from our study is the necessity for future research to examine the validity of health professional trainees’ preferences and whether these preferences align with observed behaviors. DCEs examine hypothetical scenarios and options, which has led to criticism regarding their realism and the lack of investigation into the external validity of preferences on actual behavior [67]. While previous studies on patient preferences have demonstrated that DCEs predict behavior effectively, few studies have confirmed whether these studies accurately predict outcomes, such as satisfaction, actual job choice, or residency uptake [3]. For instance, while there is literature on what drives physicians and nurses to rural areas, there is a lack of knowledge on actual choice behavior. Future research needs to understand whether preferences in location are consistent with whether physicians actually choose to work in a rural area. The same is also needed for the choice of residency or postgraduate training for physicians. Understanding whether preferences for location are associated with actual behavior has the potential to help policymakers craft policies to increase access to clinicians.

Future research should also explore the specific interactions that facilitate the integration of trainees’ preferences into curricular or programmatic changes that are associated with educational outcomes. Prior research has shown that student preferences for learning modalities (i.e., learning styles) do not predict actual student achievement and may even hinder academic performance by constraining educators’ and others’ perceptions of students’ potential academic success [68,69].

Like all reviews, this review contains limitations that might impact the conclusions that can be drawn. First, our search may not have included all HPE DCE-type studies. We attempted to mitigate this by including multiple databases, including the GIM, which covers literature from the ‘global south’, which is traditionally not included in PubMed or Web of Science databases [70]. We specifically sought out this literature to help increase the impact from those authors as well as provide that resource for other authors to search those databases, further increasing their impact. *A priori,* we also attempted to include literature in languages other than English to ensure that we captured as much of the literature as possible. Only 3 studies (one each written in Japanese, Chinese, and Portuguese) were located that were not written in English [19,33,56]. One study was evaluated by a native speaker [19], another was translated by a native speaker [56], and one was translated using Google translation [33]. Like other scoping reviews, we also did not attempt to identify whether there was publication bias. We also did not include Embase or Scopus in our literature search. Instead, we chose to search the Web of Science and EconLit databases. Prior work has shown that there is substantial overlap between Pubmed, Embase, and Scopus, potentially as high as 80% [71].

The findings of this review present the first synthesis of the DCE methodology in HPE. We found that the methodology has been used in a variety of thematic areas, highlighting its flexibility in identifying preferences not only among health learners but also among faculty. The methodology has been used mostly to identify learner preferences for their jobs and specialties but has also been used to identify preferences for clinical and didactic training. While the methodology is able to identify preferences, more methodological development is necessary to identify whether stated preferences predict actual behavior.
